# Supplementary material for: A high‐resolution 3D atlas of the spectrum of tuberculous and COVID‐19 lung lesions
Source: EMBO Mol Med. 2022 Oct 26;14(11):e16283. doi: 10.15252/emmm.202216283 (PMC9641421; doi:10.15252/emmm.202216283)
Supplement: Supplementary file 1 — Appendix [file EMMM-14-0-s003.pdf]

Appendix

A high-resolution 3D atlas of the spectrum of tuberculous and COVID-19 lung lesions

Gordon Wells[1], Joel N. Glasgow[2], Kievershen Nargan[1], Kapongo Lumamba[1], Rajhmun Madansein[3], Kameel Maharaj[3], Leon Y. Perumal[4], Malcolm Matthew[4], Robert L. Hunter[5], Hayden Pacl[6], Jacelyn E. Peabody Lever[6], Denise D. Stanford[7,8,] Satinder P. Singh[7,9], Prachi Bajpai[10], Upender Mannei[10], Paul V. Benson[10], Steven M. Rowe[7,8], Stephan le Roux[13.], Alex Sigal[1], Muofhe Tshibalanganda[12], Carlyn Wells[11], Anton du Plessis[12,14], Mpumelelo Msimang[15], Threnesan Naidoo[1,16] and Adrie J.C. Steyn[1,2,17,*]

1 Africa Health Research Institute, University of KwaZulu-Natal, Durban, South Africa
2 Department of Microbiology, University of Alabama at Birmingham, AL, USA
3 Inkosi Albert Luthuli Central Hospital and University of KwaZulu-Natal, Durban, South Africa
4 Perumal & Partners Radiologists, Ahmed Al-Kadi Private Hospital, Durban South Africa
5 Department of Pathology and Laboratory Medicine, University of Texas Health Sciences Center at Houston, Houston, TX, USA
6 Medical Scientist Training Program, University of Alabama at Birmingham, AL, USA
7 Department of Medicine, University of Alabama at Birmingham, AL, USA
8 Cystic Fibrosis Research Center, University of Alabama at Birmingham, AL, USA
9 Department of Radiology, University of Alabama at Birmingham, AL, USA
10 Department of Pathology, University of Alabama at Birmingham, AL, USA
11 CT Scanner Facility, Central Analytical Facilities, Stellenbosch University, South Africa
12 Research group 3D Innovation, Physics Department, Stellenbosch University, South Africa
13 Bruker Belgium NV, Kontich, Belgium
14 Object Research Systems, Montreal, Canada
15 Department of Anatomical Pathology, National Health Laboratory Service, Inkosi Albert Luthuli Central Hospital, Durban, South Africa
16 Department of Laboratory Medicine & Pathology, Walter Sisulu University, Eastern Cape, South Africa
17 Centers for AIDS Research and Free Radical Biology, University of Alabama at Birmingham, AL, USA

Corresponding Author

*Adrie JC Steyn, PhD

Email: asteyn@uab.edu or adrie.steyn@ahri.org

This Appendix PDF file includes:

1. Appendix Figs S1 – S15, pg 2-18

3. Appendix Tables S1 – S2, pg 19-23

Appendix Figures

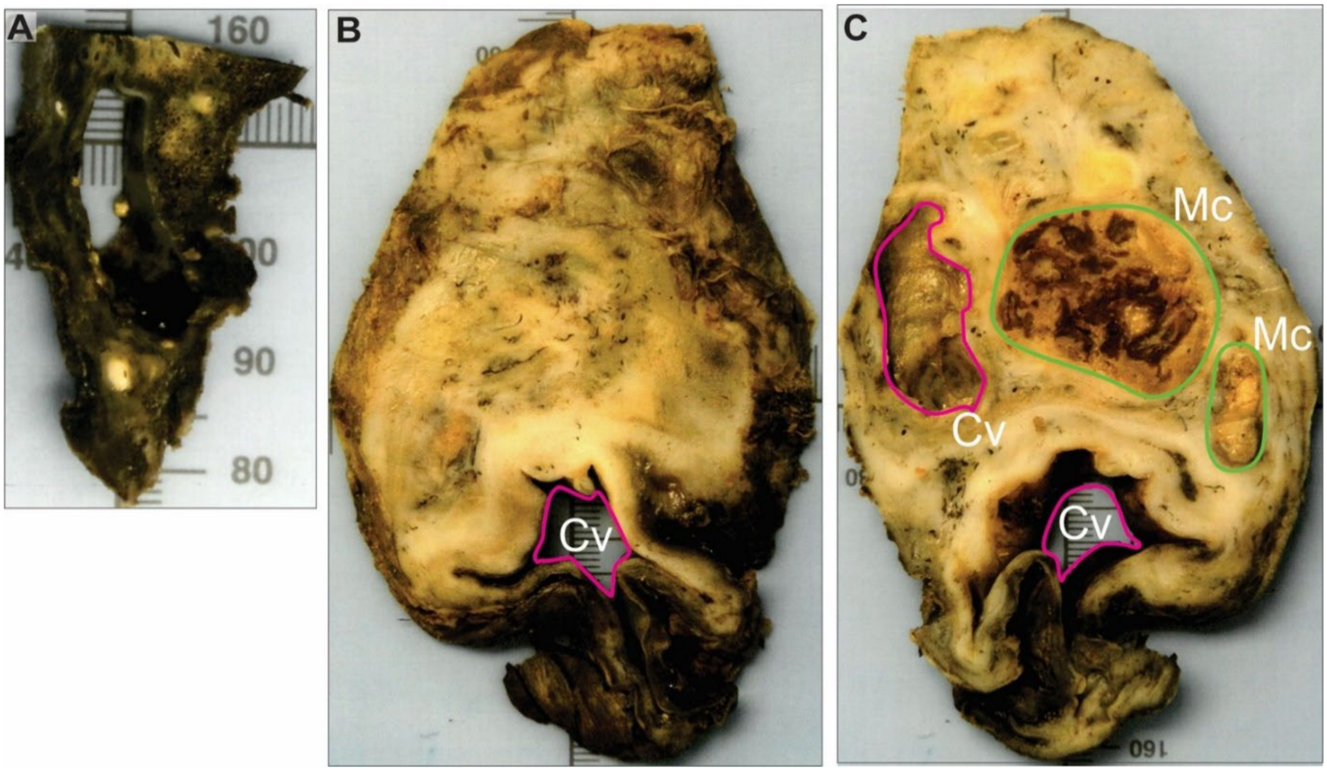

Appendix Fig S1. Gross images of lung tissue used for μ/n/clinical-CT imaging. (A) Sample A, as shown in Figure 1E, exhibiting cavitation and calcification or necrotic lesions. (B, C) Sample B, as shown in Figure 1N, exhibiting two cavities (Cv; purple outline) and two mycetomas (Mc; green outline).

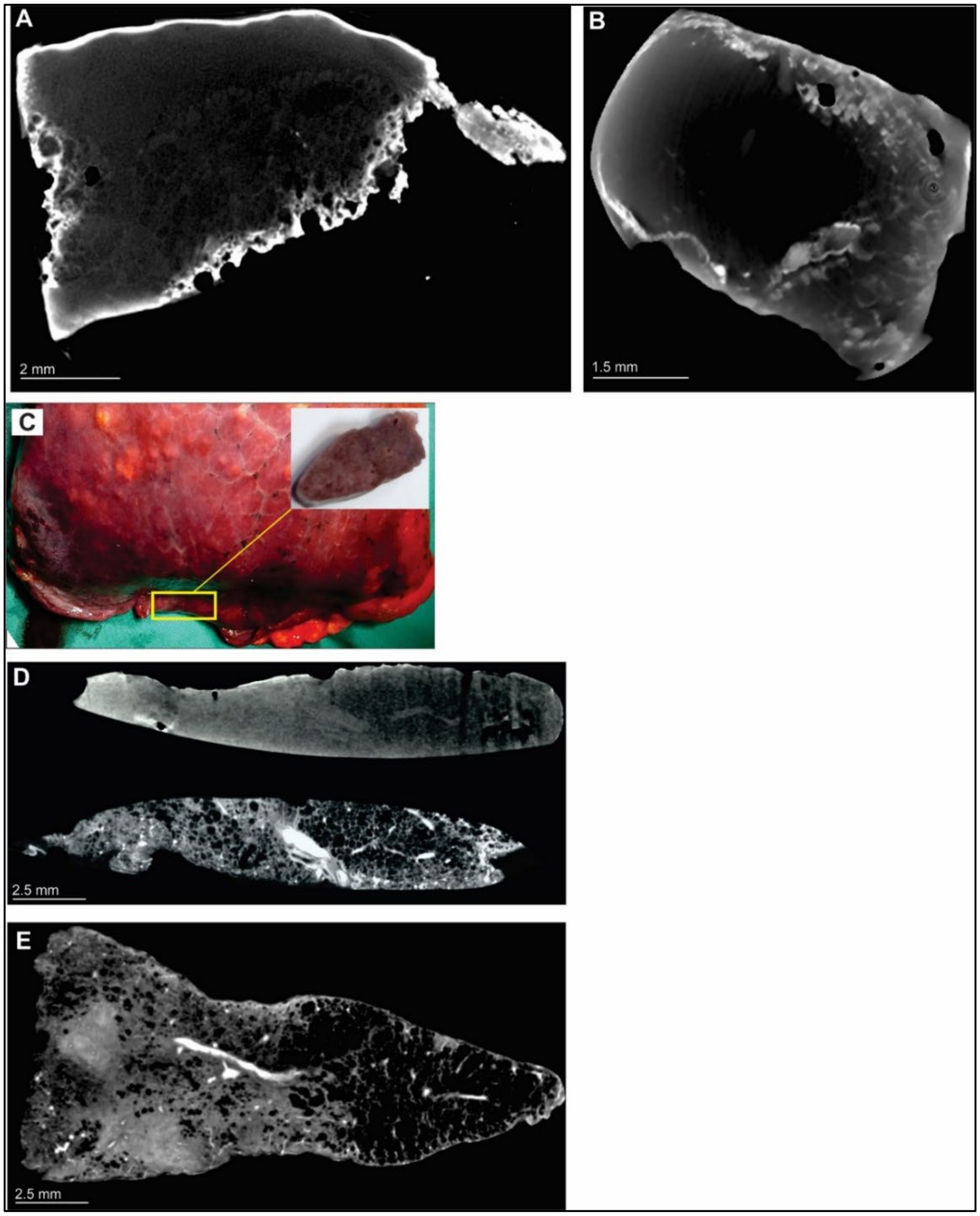

Appendix Fig S2. Optimization of contrast staining using eosin and iodine. (A) Staining of uninvolved tissue from a TB patient (Sample E) with alcohol-soluble eosin. (B) Staining of caseous necrotic tissue with alcohol-soluble eosin (Sample F). (C) Samples D and G (inset), partially healthy tissue from TB lung. (D) Contrast staining of partially healthy/uninvolved tissue (Sample D) with Lugol's solution/iodine before (top) and after (bottom) staining. (E) Contrast staining with iodine of an uninvolved tissue sample adjacent to Sample D (Sample G), demonstrating a lack of septa similar to Sample D.

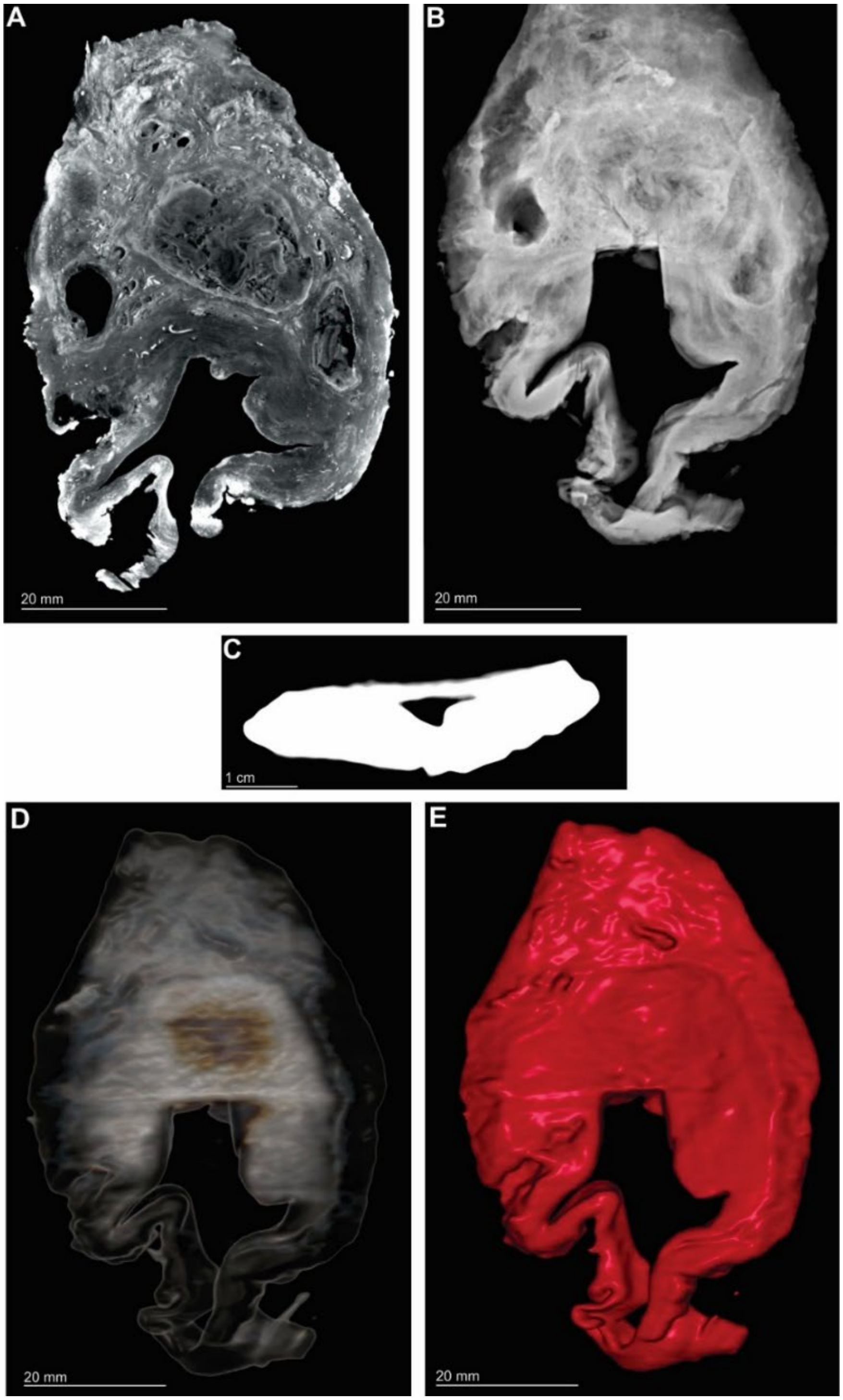

Appendix Fig S3. Enlarged comparison of µCT, HRCT and SXT scans of cavitation and mycetomas. Enlarged versions of selected panels and raw HRCT for Sample B from Figure 1. (A) µCT slice. (B) Soft X-ray scan. (C) Unprocessed transverse HRCT image. A series of these images is used to reconstruct a volume that can generate images such as in A, B and D. (D) Onco-liver HRCT preset. (E) "Bone-metal" HRCT preset.

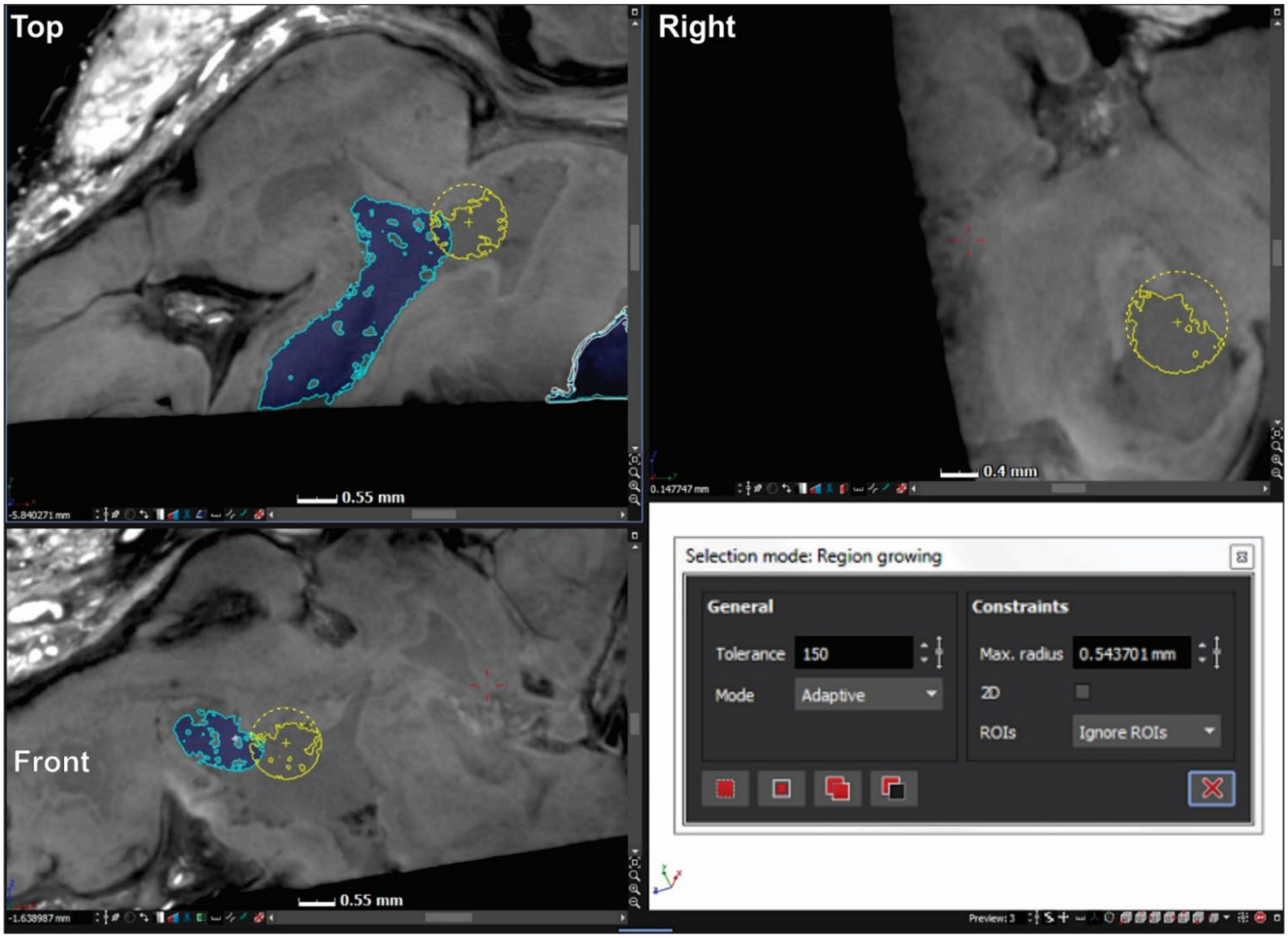

Appendix Fig S4. Manual segmentation by region growing. An example of using the region growing tool in VGStudio Max. An already segmented region (blue) is extended by selecting voxels within a user-defined sphere (yellow dotted line) that satisfy the desired tolerance criteria (yellow solid line). Paraphrasing the VGStudio manual, “Adds voxels to the growing region if they are connected to the region and their gray values do not differ by more than the Tolerance value of 150 from the average gray value of the local voxels of the growing region around the candidate voxel”. Accurate segmentation requires constant manual adjustment of the various parameters and experimentation with different selection modes.

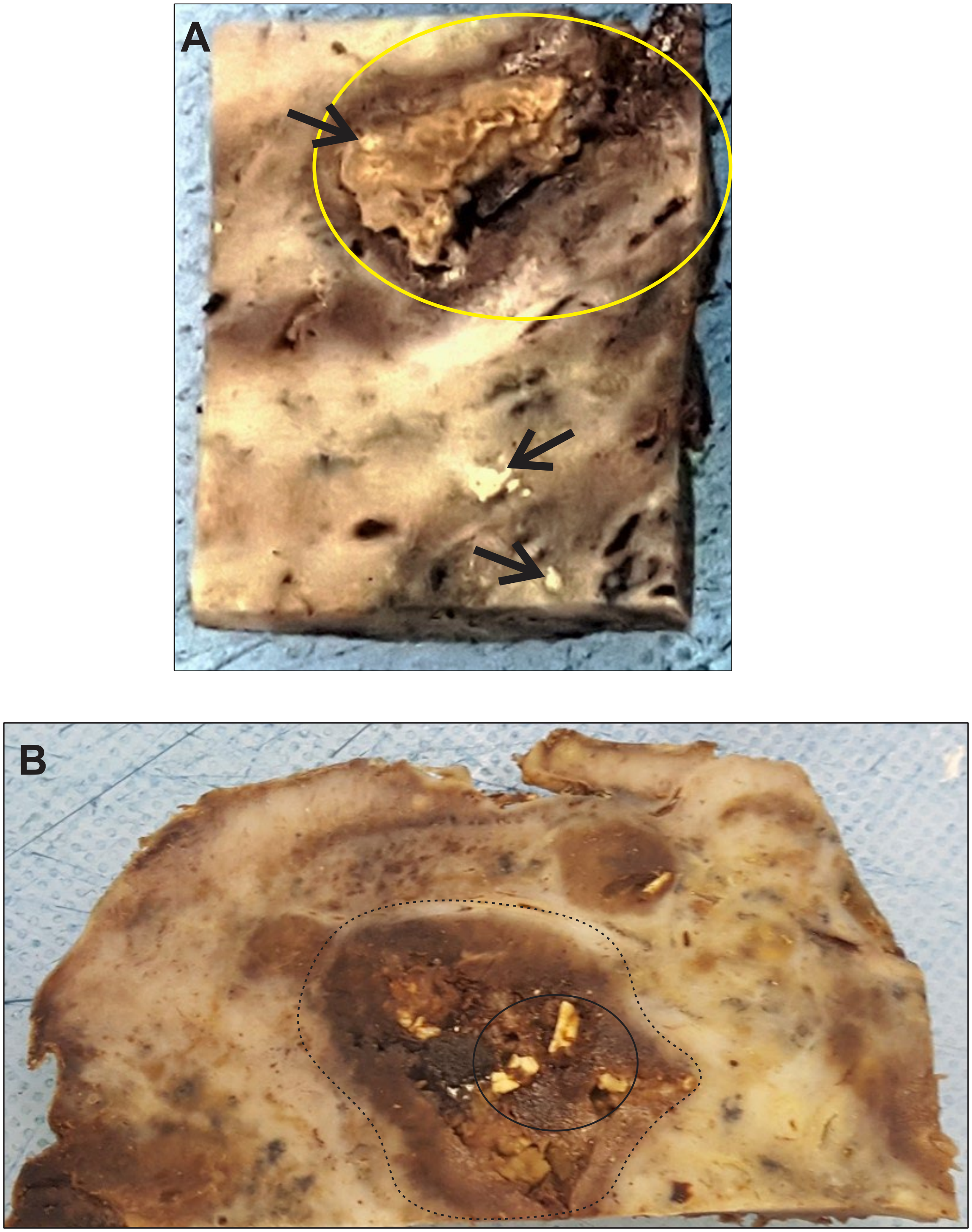

Appendix Fig S5. Gross images of FFPE samples. (A) Sample H exhibiting calcification (black arrows) and mycetoma (yellow circle); scanned as an FFPE block. (B) Sample I exhibiting calcification (black circle) and mycetoma (black dotted outline); scanned as an FFPE block.

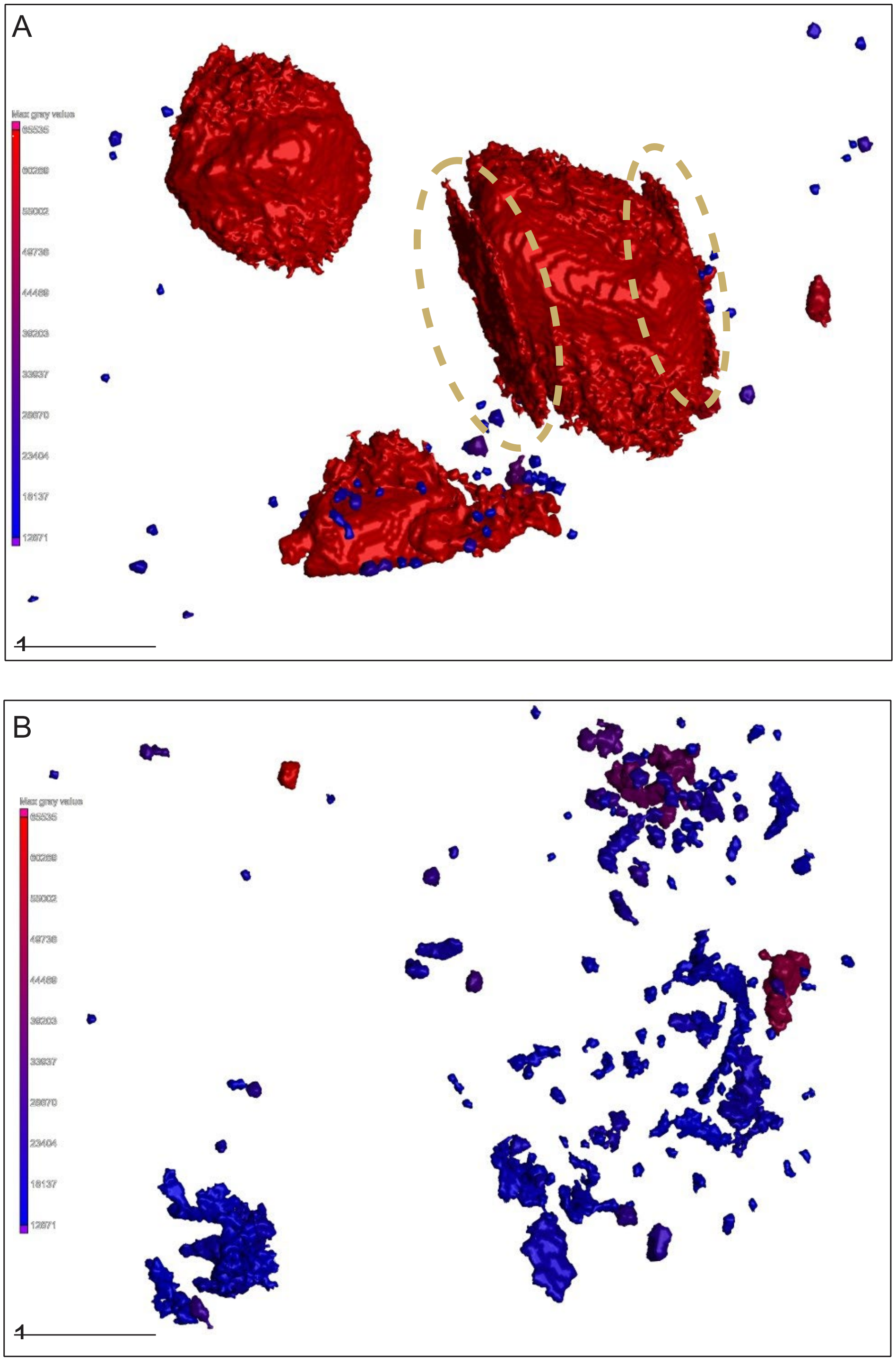

Appendix Fig S6. Heterogeneity of high-density regions and calcification. High-density regions (density > 11502) were used to generate ROI revealing calcification. The segmentation also includes halo artefacts (regions within dotted lines).

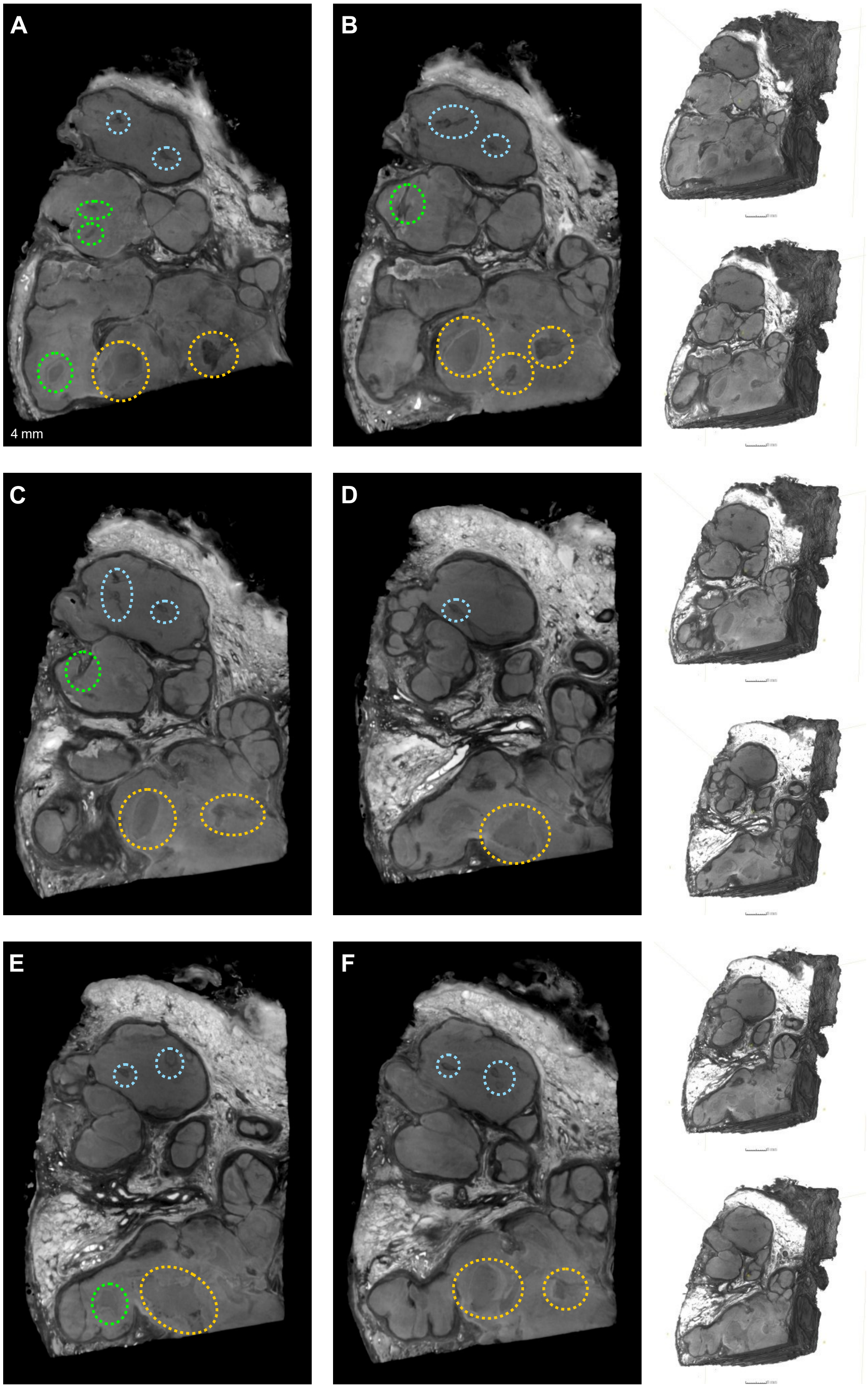

Appendix Fig S7. Anomalies within necrotic lesions. (A-F) Sequential slices adjacent to μCT slice shown in Fig 6A highlighting evolution of anomalies within lesions. Regions circled in orange and blue were segmented (Fig 6C-G). Green circled regions were not segmented.

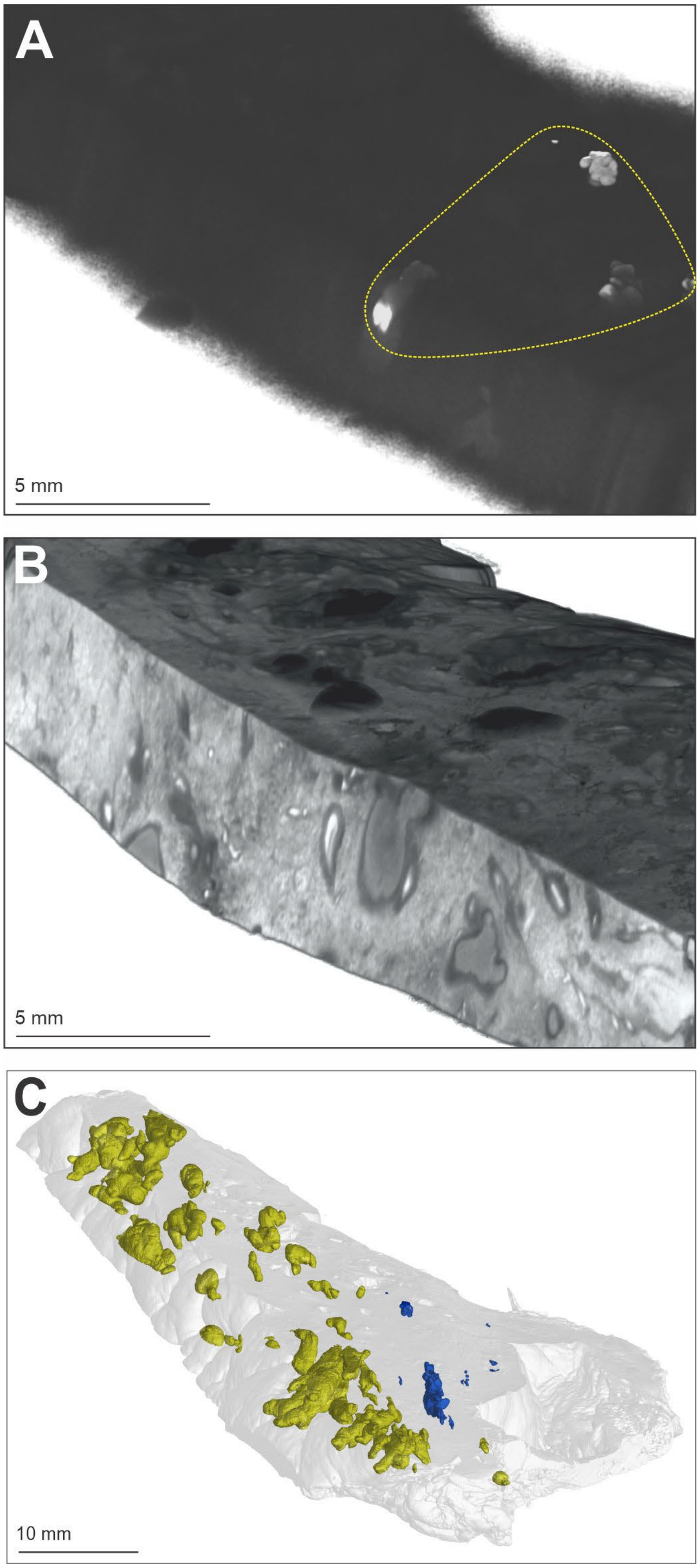

Appendix Fig S8. Spatial organization of necrotic lesions and calcification. Overlay of scan before (A; calcium crystals encircled in yellow) and after (B) staining with iodine. (C) 3D rendering of necrotic lesions (yellow) and calcium crystals (blue).

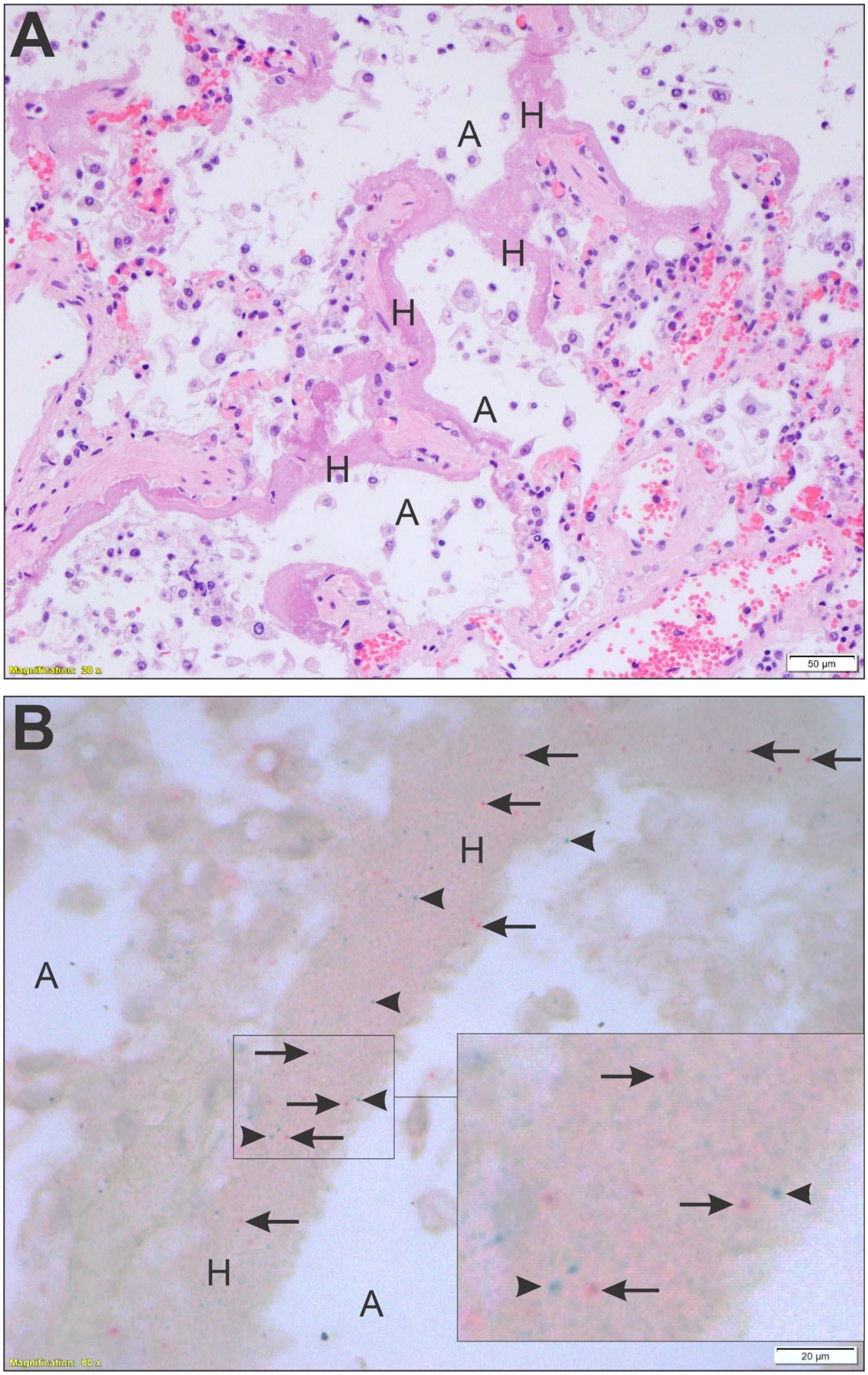

Appendix Fig S9. Diffuse alveolar damage in COVID-19 tissue. (A) H&E stain of diffuse alveolar damage, A: alveolar space and H: hyaline membrane. (B) RNA-Scope of SARS-CoV-2 with anti-sense RNA probe (black arrows/red signal) and sense probe (arrow heads, turquoise signal). A; alveolar space, H; hyaline membrane. 60x magnification. Inset; high-power image of boxed area.

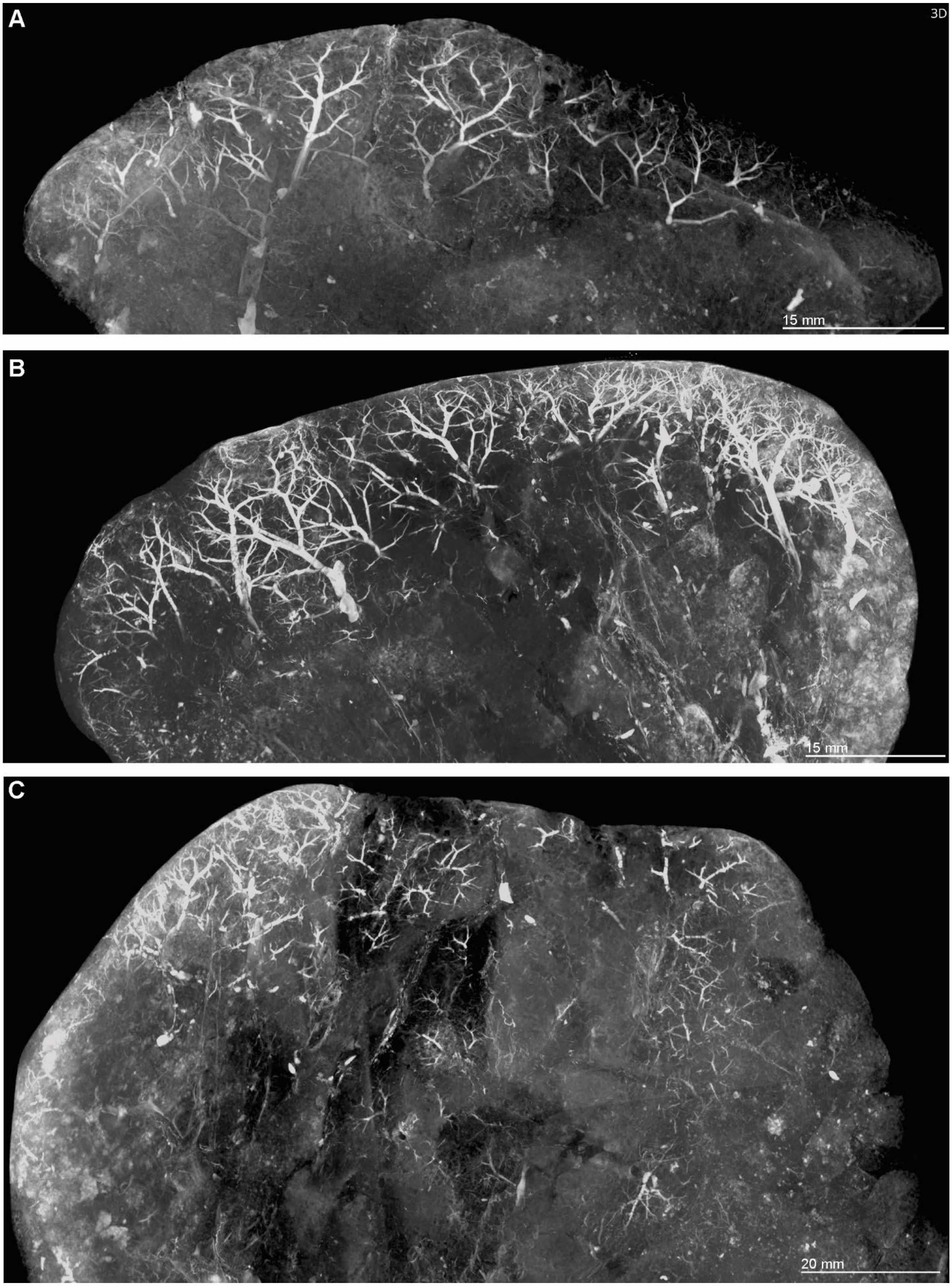
A
3D
15 mm
B
15 mm
C
20 mm

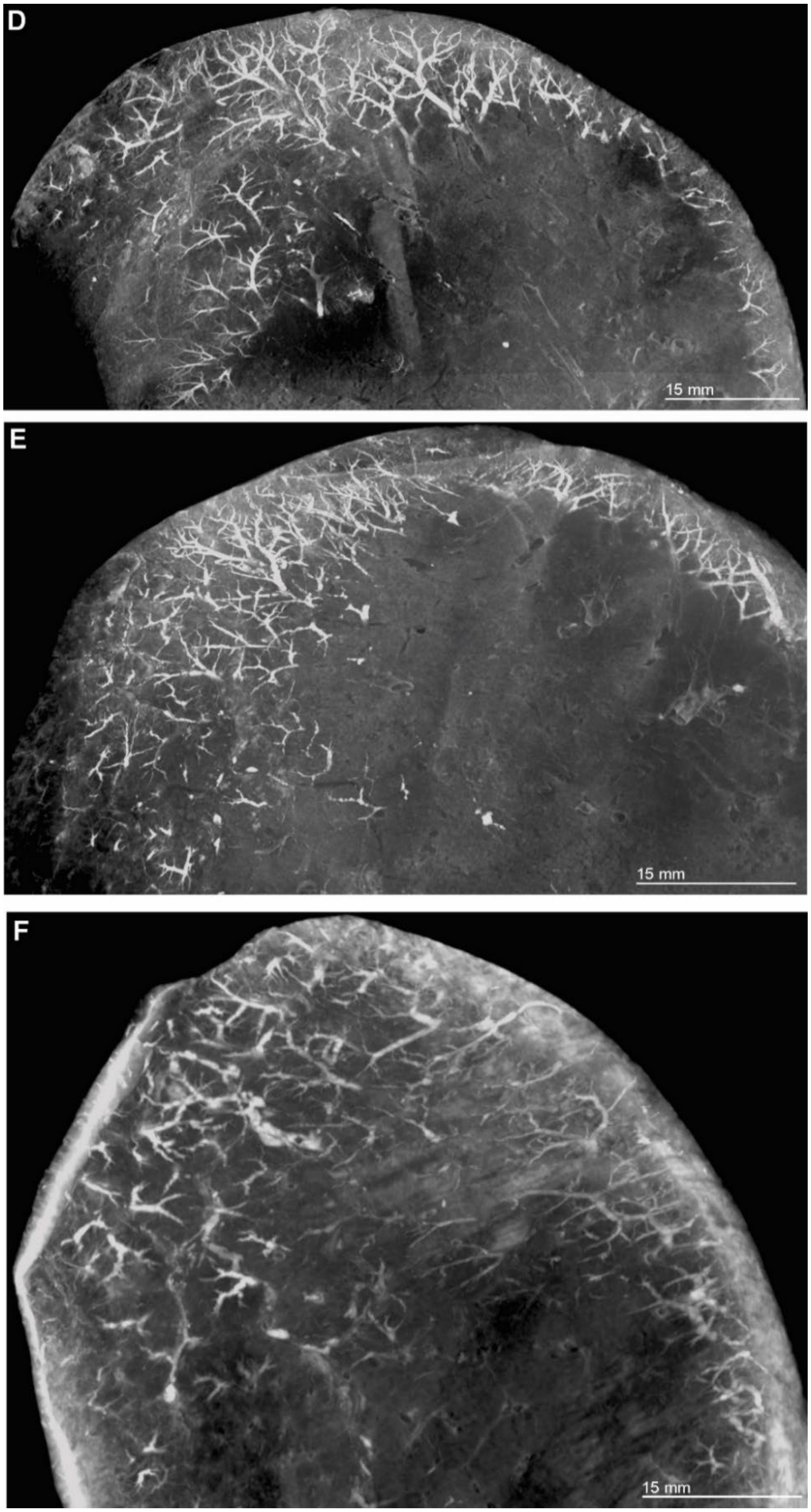
D
15 mm
E
15 mm
F
15 mm

Appendix Fig S10. Medium power μCT images showing peripheral vasculature. (A-F) Maximal-intensity images of an upper right lung lobe divided into six slices reveal more intact vasculature near the periphery.

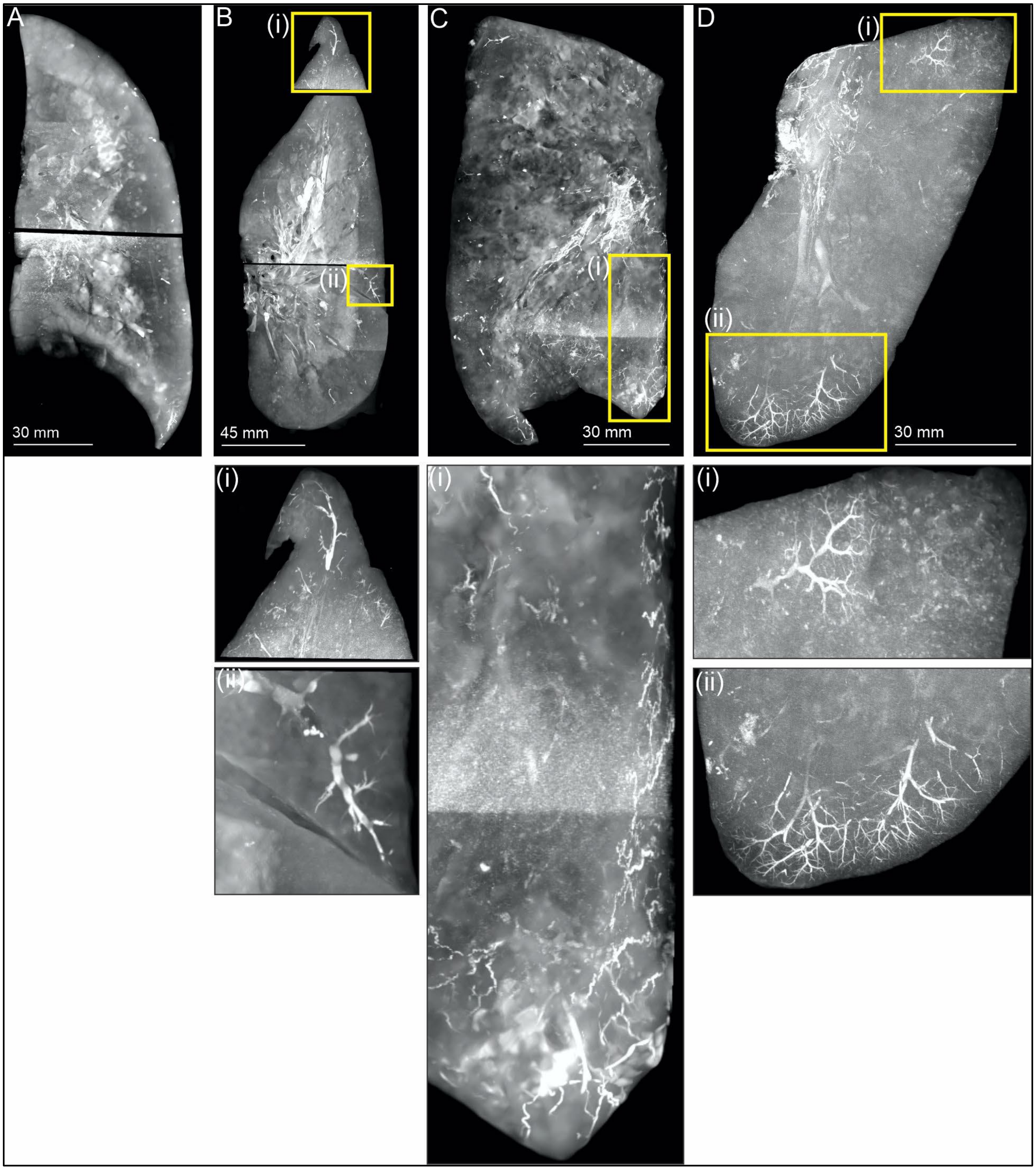

Appendix Fig S11. µCT of postmortem COVID-19 lung slices. Maximal-intensity images of post-mortem lung slices from four decedents. Note the absence of peripheral vasculature in (A), but the presence of vasculature in B, C and D to lesser and greater extent. (B-D) Yellow boxed areas showing peripheral vasculature are shown at higher magnification below the main image. Some samples were too large to be scanned whole, therefore partial sequential scans were combined. Stitching during reconstruction was not available, therefore scans were combined in VGStudio Max, resulting in edge artefacts and incomplete joins.

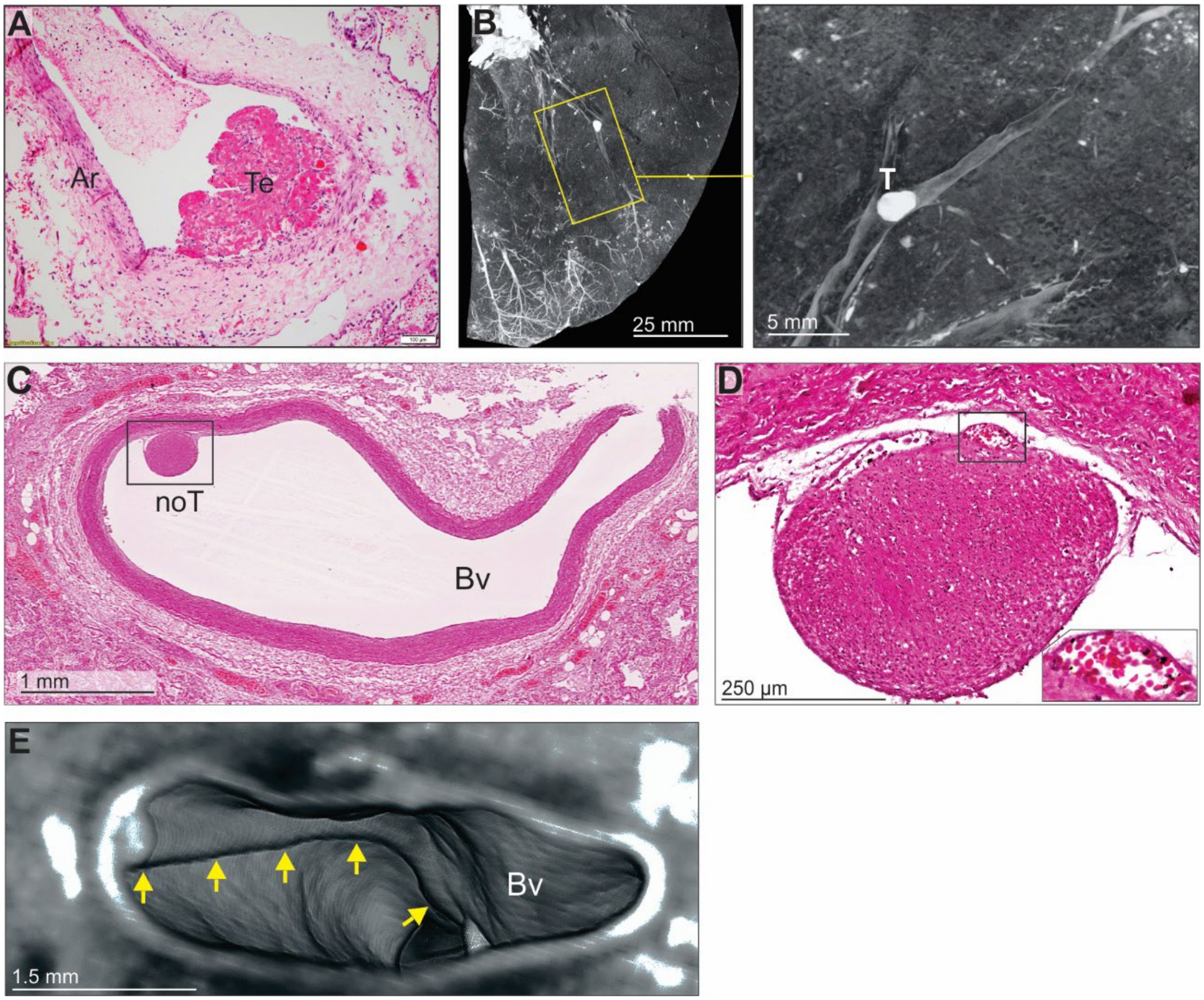

Appendix Fig S12. Vascular occlusions in COVID 19 lungs. (A) H&E of pulmonary thromboembolism (Te). (B) Occlusion at the branch point of two blood vessels; maximal intensity projection. (C) H&E of a non-organizing occlusion (noT). (D) Magnified view of noT in (C). (E) µCT of the noT from (C, D) reveals a long tubular structure (yellow arrows) within the blood vessel (Bv).

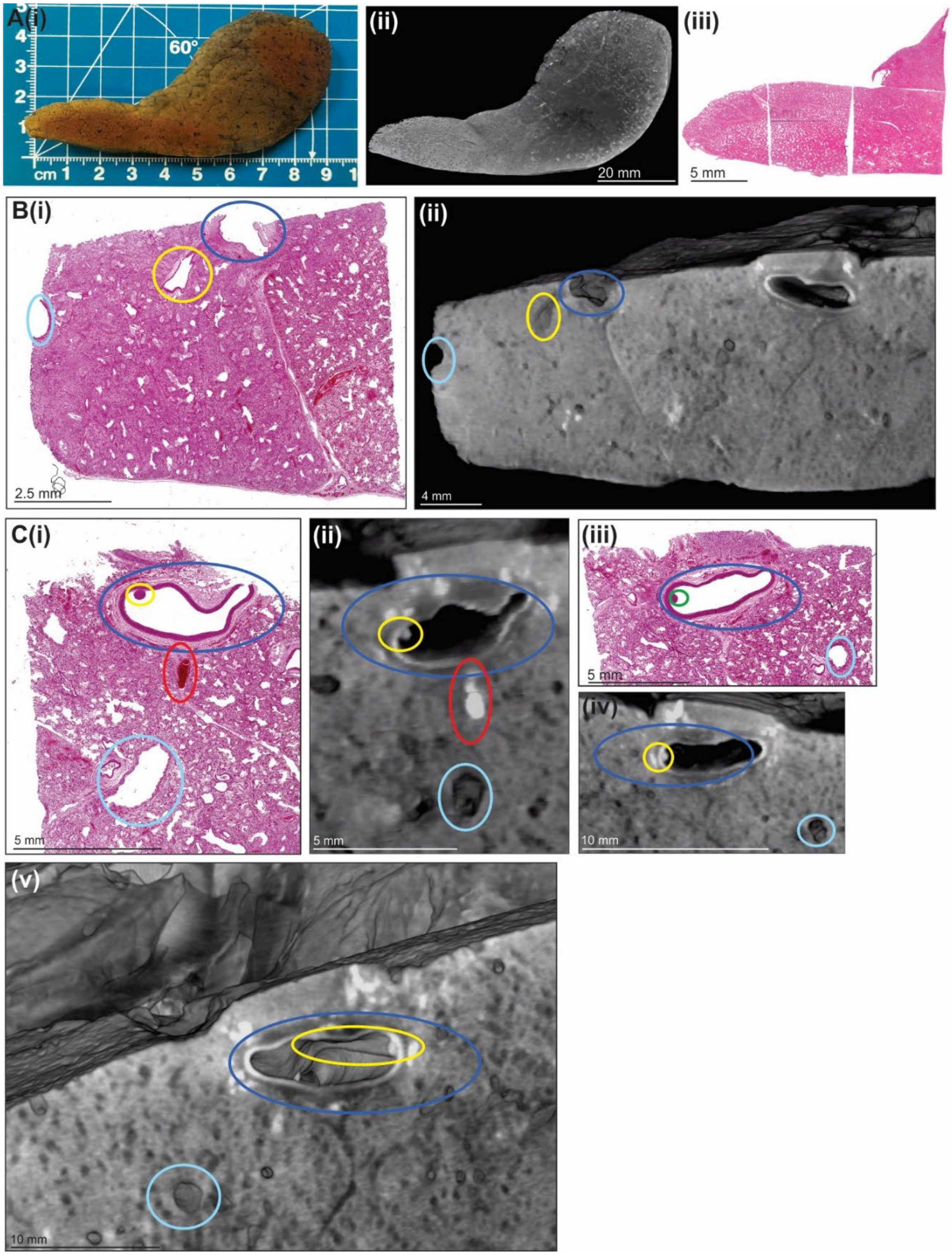

Appendix Fig S13. μCT and matched histology of COVID-19 lung. (A) (i) Gross image of smallest slice of upper lobe from right lung. (ii) μCT of i. (iii) H&E histology of i. (B) Matched H&E histology (i) and μCT (ii) of a larger slice from the same lobe. (C) Matched H&E histology (i, iii) and μCT (ii, iv, v) of an adjacent region to B. Matched features are indicated with same-color circles.

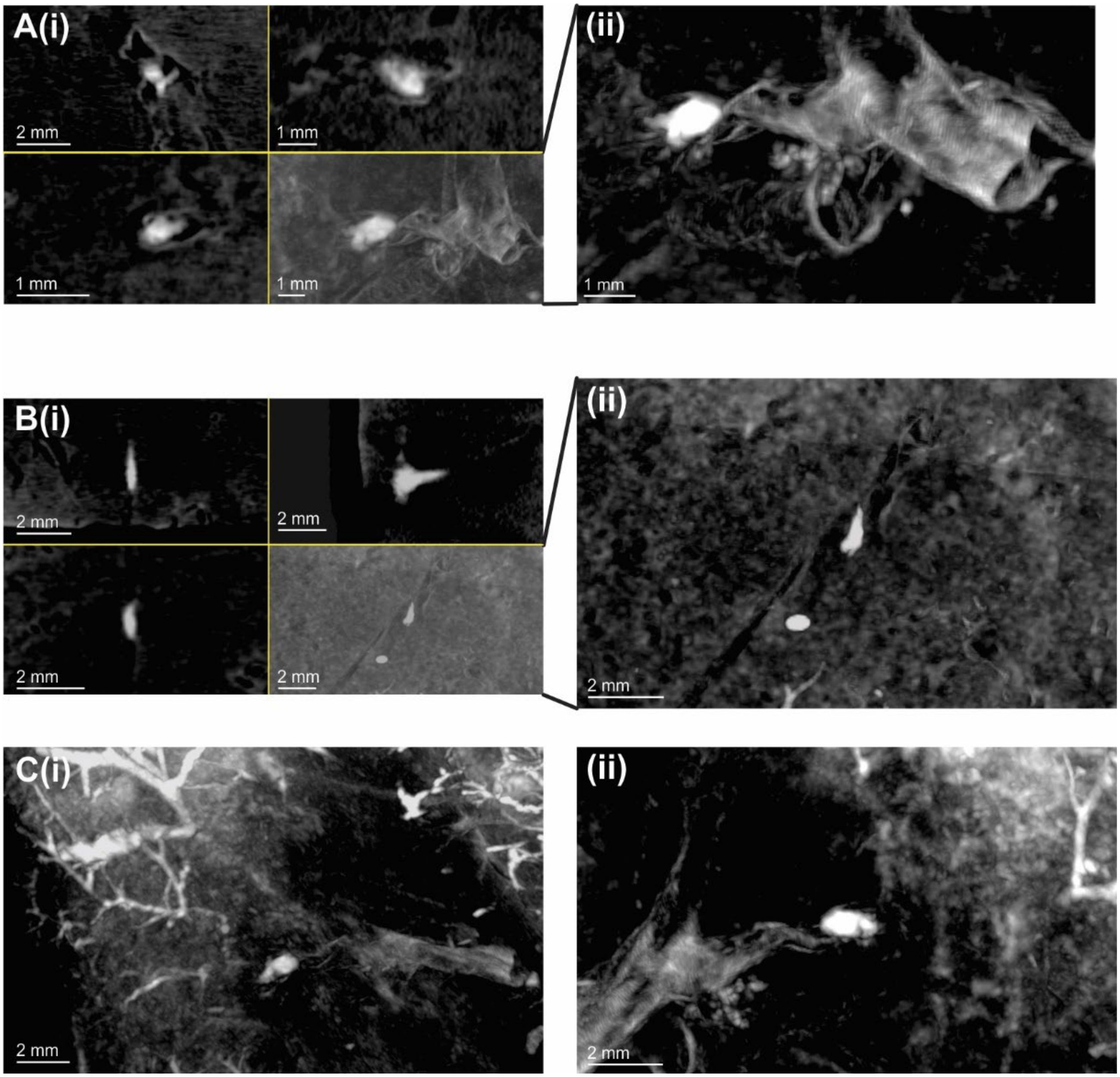

Appendix Fig S14. Occlusions viewed by µCT. Maximum intensity projects of three separate occlusions (A, B, C, respectively). (A, B) Two separate occlusions. Left column (i): occlusion viewed from three orthogonal directions (green lines: top right and left, bottom left) and as maximal intensity projection (bottom right). Right column (ii): Enlarged view of maximal intensity projection from (i / bottom right) panel. (C) Two views of a third occlusion, viewed from approximately opposite directions.

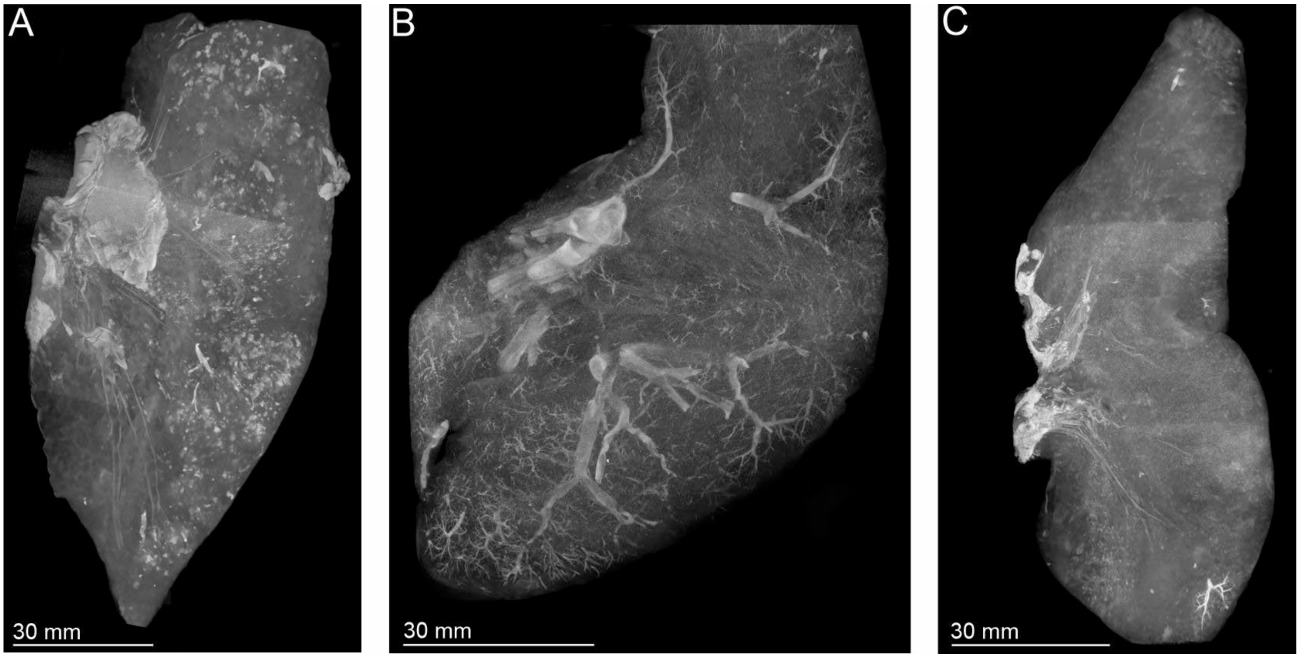

Appendix Fig S15. μCT of non-tuberculosis and non-COVID 19 lungs. (A) Sample 12, (B) Sample 14 and (C) Sample 13. See Appendix Table S1 for clinical details.

Appendix Table S1. Clinical characteristics of human subjects.

#	Age	Sex	Type	Macroscopic/microscopic features, pulmonary findings and other comments	Type of resection/History
1	37	F	TB	Lung specimen weighed 72 g, bronchiectasis and fibrosis are noticed. Multiple tubercles (1-10 mm) are noted, lung tissue shows distorted architecture by confluent necrotizing and non-necrotizing granulomatous inflammation. Acid-fast bacilli positive, Langhans giant cells are present, dilated blood vessels are seen, alveolar spaces are filled with eosinophilic material and hemorrhage.	RUL lobectomy
2	15	F	TB	Lobectomy specimen weighing 48 g; multiple tubercles are noted on cut section. Sections of tubercles confirmed active necrotizing granulomas, acid-fast bacilli are seen, vasculitis and interstitial fibrosis are present.	RUL lobectomy
3	28	M	TB	Lobectomy specimen weighing 132 g; upper lobe appears shrunken, bronchiectasis and fibrosis noted, tubercles appear irregular in shape ranging from 1-3 mm, calcified foci are confirmed, focal tuberculous scars and fibrocaseous foci are noted.	LUL lobectomy
4	33	F	TB	Lung tissue weighing 192 g, shrunken upper lobe collapsed with bronchiectasis and fibrosis, tubercles (1-6 mm) appear irregular in shape, irregular cavity is noted, granulation tissue is prominent, aspergillosis, paucibacillary.	Left pneumonectomy
5	50	M	TB	Specimen weighed 606 g, the lung is bronchiectatic and a cavity is noted. Cavity wall lined by chronic suppurative inflammation, granulation tissue and foamy histiocytes is observed, aspergilloma is present, hemorrhage, or oedema and organizing pneumonia are seen.	Right pneumonectomy
6	65	F	TB	Lung tissue weighing 318 g, tubercles (1-14 mm) with bronchopneumonia are noted. Sections demonstrate extensive granulomatous inflammation and tubercles, acid-fast bacilli are present, interstitial fibrosis and areas of gross scarring are noted.	Right pneumonectomy
7				Healthy lung tissue from lung cancer patient	
8	54	M	COVID-19	Pulmonary edema. Emphysematous changes are present. Changes of organizing diffuse alveolar damage are present including abundant intra-alveolar macrophages, hyperplasia of type II pneumocytes (with occasional squamous metaplasia), fibroblastic proliferation organizing hyaline membranes and acute hyaline membranes. There is a moderate interstitial mononuclear cell infiltrate present throughout the sections. Intra-alveolar neutrophils are present in scattered alveolar spaces especially in the right lower lobe and left lower lobe a focal organizing pulmonary thromboembolus is present in the left upper lobe.	End stage renal disease s/p kidney transplant complicated by MSSA and *Serratia pneumonia* and CMV viremia, BMI: 31.6.
9	36	M	COVID-19	Acute and organizing pneumonia and acute and organizing DAD. There appears to be thrombi in small vessels and in the left lower lobe a larger artery has what looks more like a pulmonary thromboembolism.	BMI: 41, MSSA pneumonia, type II diabetes, sepsis *E. faecium* and *P. aeruginosa.*
10	68	F	COVID-19	Changes of organizing diffuse alveolar damage are present including cellular and acellular hyaline membranes, intra-alveolar fibroblastic proliferation, intra-alveolar macrophages, and activation of type II pneumocytes with squamous and bronchiolar metaplasia. Moderate diffuse interstitial lymphocytic infiltrate is present throughout the sections. Occasional alveolar spaces show intra-alveolar neutrophils admixed with fibrin. Multifocal occlusive and partially occlusive organizing thromboemboli are present throughout the sections. Emphysematous changes are present.	HTN, DM, BMI: 45, emphysema

				Pulmonary edema is present. Rare subepithelial microthrombi are present in a cartilaginous bronchus.	
11	30	F	COVID-19	Focal consolidation with abscess formation and possible focal infarction, most consistent with bacterial pneumoni	DM, MRSA and *Protues mirabilis* pneumonia leading to septic shock. BMI: 41
11	67	M	COVID-19	Pulmonary edema. Right lung weighs ~1.4 kg. Abundant intra-alveolar and interstitial fibroblastic proliferation with abundant macrophages and other mononuclear cells were noted. Few areas of recognizable alveolar air spaces are present, likely due to the proliferation of fibroblasts, macrophages, and type II pneumocytes. Overall, the lung displayed a much proliferative and solid appearance.	Post-mortem
13	50	M	Control	Prominent pleural cobblestoning and parenchymal fibrosis, most prominent in subpleural areas	Interstitial lung disease consistent with scleroderma on steroids, BMI: 30.1
14	56	F	Control	Prominent congestion without obvious consolidation (2,360g). Pleural adhesions among the lung lobes, more prominent in the right lung, without adhesions. Abundant blood in airways, likely aspirated	Hepatocellular carcinoma, NASH cirrhosis, obesity BMI: 33.6, COPD, esophageal varicies, septic shock and anasarca
15	75	F	Control	Bilateral pulmonary thromboemboli of lungs. Edema, vascular congestion.	Polycythemia vera, autoimmune motor neuropathy, quadriparesis, multiple thrombi of many vessels including cerebral veins and pulmonary arteries.

Pulmonary thromboembolism (PTE), right upper lobe (RUL), lower upper lope (LUL), body mass index (BMI), cytomegalovirus (CMV), methicillin sensitive *Staphylococcus aureus* (MSSA), hypertension (HTN), diabetes mellitus (DM), diffuse alveolar damage (DAD).

Appendix Table S2. Samples and scanning settings for μCT and nCT.

Sample	Dimensions	Target	Instrument	Scan time (s)	Averaging	Skip	Voxel size (μm)	Voltage (kV)	Current (μA)	Images	Pathology	Preparation	Figures
**A**	2.5 × 4 × 0.8 cm	W	HRCT	<10 min	-	-	-	-	-		Cavitation	Formalin fixed only	1G, H
		Mo/Rh	SXT	<5 min									1F
**B**	11 × 7 × 1.5 cm	W	HRCT	<10 min	-	-	-	-	-		Mycetoma & cavitation	Contrast stained with I_2	1R-U S3C-E
		Mo/Rh	SXT	<5 min	-	-	-	-	-				1P, Q S3B
		W	μCT	3000	5	1	60.0	160	200	3000			1O 4E-G S2 S3A
**C**	6 × 5 × 3 mm	W	nCT	5400	1	0	4.5	60	280	5400	Healthy (from cancerous lung)	Contrast stained with I_2	2A, C
**D**		Mo	nCT	3600	1	0	16.0	60	300	3600	Partially healthy (from TB lung)	Contrast stained with I_2	3D, E
	19 × 5 × 3 mm	Mo	nCT	5400	1	0	4.1	60	280	5400			3B, C S1
		W	μCT	2000	5	1	20.0	80	250	2000			3A S2D bottom
		W	μCT	2000	5	1	20.0	80	250	2000		Unstained	S2D top
**E**	6.5 × 5 × 11 mm	W	μCT	2400	2	1	15.0	60	300	2400	Uninvolved/healthy tissue	Contrast stained with alcolhol soluble eosin	S2A
**F**	14 × 10 × 18 mm	W	μCT	2400	2	1	15.0	60	300	2400	Caseous necrosis	Contrast stained with alcohol solbule eosin	S2B
**G**	18 × 9 × 4 mm	Mo	nCT	3600	1	0	10.0	70	220	3600	Uninvolved/healthy tissue	Contrast stained with I_2	S2E
**H**	25 × 18 × 4.5 mm	W	μCT	2000	3	1	26.8	60	430	2000	Mycetoma	Wax block. No contrast staining	4B
		W	μCT	2400	2	1	12.0	50	240	2400		Wax removed. No contrast staining	4F,I,J S6
**I**	30 × 29 × 4.5 mm	W	nCT	3600	1	0	8.5	60	280	3600	Calcification	Wax block. No contrast staining	4O
		W	μCT	2000	3	1	40.0	100	100	3600	Calcification		4G,H
**J**	20 × 15 × 4 mm	W	μCT*	16231	5	0		60	149	1801	Caseous necrosis	Unstained, FFPE block	5C-G
**K**	18 × 13 × 9 mm	W	μCT	2800	5	1	15.0	80	400	2800	Caseous necrosis	Contrast stained with I_2	6A, C-G S7
**L**	14 × 1.5 × 6 cm	W	μCT	3000	5	1	52..0	160	260	3000	Severe caseous necrosis and consolidation across lung	Contrast stained with I_2	6I-K S8
**M1**	16 × 7 × 1.5 cm	W	μCT	3200	1	0	70	160	220	3200	COVID 19	Contrast stained with I_2	7C S10A-C
**M2**	18 × 7.5 × 1 cm	W	μCT	3200	3	1	65.2	160	220	3200	COVID 19	Contrast stained with I_2	7C, 8A,B,C,G S10A-C S12A S13 B,C,
**M3**	16/3 × 11/0 × 1.5 cm	W	μCT	3200	1	0	70	160	220	3200	COVID 19	Contrast stained with I_2	7C S10A-C
**M4**	13 × 8.5 × 1 cm	W	μCT	3200	1	0	70	160	220	3200	COVID 19	Contrast stained with I_2	7C S10D-F
**M5**	13 × 8 × 1 cm	W	μCT	3200	1	0	70	160	220	3200	COVID 19	Contrast stained with I_2	7C S10D-F

													S14
**M6**	9 × 4 × 1 cm	W	µCT	3200	1	0	70	160	220	3200	COVID 19	Contrast stained with I_2	7C<br>S10A, S10D-F
**N**	12 × 5 × 11.5 cm	W	µCT	3000	2	0	50	160	220	3000	COVID 19	Contrast stained with I_2	6D
**O**	22 × 7 × 1.5 cm		µCT (UAB)	480	4	0	80	55	190	1440	COVID 19	Contrast stained with I_2	S12A
**P**	16.5 × 6.5 × 2 cm		µCT (UAB)	480	4	0	80	55	190	1440	COVID 19	Contrast stained with I_2	S12B
**Q**	15 × 8 × 1 cm		µCT (UAB)	480	4	0	80	55	190	1440	COVID 19	Contrast stained with I_2	S12C
**R**	13 × 5.5 × 1.5 cm		µCT (UAB)	480	4	0	40	55	190	1440	COVID 19	Contrast stained with I_2	S12D
**S**	14 × 6 × 2 cm		µCT (UAB)	480	4	0	80	55	190	1440	Interstitial lung disease	Contrast stained with I_2	S15A
**T**	10 × 6.5 × 1.5 cm		µCT (UAB)	480	4	0	80	55	190	1440	PTE, COPD	Contrast stained with I_2	S15B
**U**	12 × 6 × 2 cm		µCT (UAB)	480	4	0	80	55	190	1440	COPD	Contrast stained with I_2	S15C

TB samples are listed first. Tungsten (W), Rhodium (Rh), Molybdenum (Mo), micro-computed tomography (µCT), nano-computed tomography (nCT), soft-X-ray tomography (SXT, mammography), pulmonary thromboembolism (PTE), chronic obstructive pulmonary disease (COPD). See Supplementary Materials and Methods for instrument details. *Sample was scanned with a Bruker 2214 X-ray microscope.
