# Supplementary material for: A high‐resolution 3D atlas of the spectrum of tuberculous and COVID‐19 lung lesions
Source: EMBO Mol Med. 2022 Oct 26;14(11):e16283. doi: 10.15252/emmm.202216283 (PMC9641421; doi:10.15252/emmm.202216283)
Supplement: Supplementary file 2 — Movie EV1 [file EMMM-14-0-s004.zip › EMM-2022-16283-V3-Movie_EV1/Movie EV1.docx]

## Movie EV1. Vasculature and airways in an uninvolved TB infected lung (Sample D).

Segmentation of vasculature (red) and airway (yellow). The vasculature forms a disconnected volume, compared to the single volume observed when segmenting vasculature in healthy lung (Sample C) from a lung cancer patient (Fig 2C).
