# Supplementary material for: A high‐resolution 3D atlas of the spectrum of tuberculous and COVID‐19 lung lesions
Source: EMBO Mol Med. 2022 Oct 26;14(11):e16283. doi: 10.15252/emmm.202216283 (PMC9641421; doi:10.15252/emmm.202216283)
Supplement: Supplementary file 3 — Movie EV2 [file EMMM-14-0-s006.zip › EMM-2022-16283-V3-Movie_EV2/Movie EV2.docx]

## Movie EV2. Clipping of X-ray intensity in sample with cavities and mycetoma (Sample B).

The cavity lies in front of the mycetoma. As the clipping plane proceeds past the cavity a comparative lack of vasculature can be observed in the cavity wall, which is consistent with histopathology analysis (Fig 4H, I). A complex system of folds can be observed within the mycetoma, surrounding be a mixture of intact and damaged vasculature including blood pools.
