# Supplementary material for: A high‐resolution 3D atlas of the spectrum of tuberculous and COVID‐19 lung lesions
Source: EMBO Mol Med. 2022 Oct 26;14(11):e16283. doi: 10.15252/emmm.202216283 (PMC9641421; doi:10.15252/emmm.202216283)
Supplement: Supplementary file 4 — Movie EV3 [file EMMM-14-0-s009.zip › EMM-2022-16283-V3-Movie_EV3/Movie EV3.docx]

## Movie EV3. Thresholding of mycetoma from FFPE block (Sample F).

Gradually increasing the iso-value of X-ray intensity within the mycetoma eventually reveals calcium nodules of various shapes and densities. No contrast staining was applied to this sample.
