# Supplementary material for: A high‐resolution 3D atlas of the spectrum of tuberculous and COVID‐19 lung lesions
Source: EMBO Mol Med. 2022 Oct 26;14(11):e16283. doi: 10.15252/emmm.202216283 (PMC9641421; doi:10.15252/emmm.202216283)
Supplement: Supplementary file 5 — Movie EV4 [file EMMM-14-0-s001.zip › EMM-2022-16283-V3-Movie_EV4/Movie EV4.docx]

## Movie EV4. Clipping of X-ray intensity through a calcium nodule from a FFPE block (Sample G).

Clipping through a large calcium nodule from an FFPE block reveals lacuna. No contrast staining was applied to this sample.
