# Supplementary material for: A high‐resolution 3D atlas of the spectrum of tuberculous and COVID‐19 lung lesions
Source: EMBO Mol Med. 2022 Oct 26;14(11):e16283. doi: 10.15252/emmm.202216283 (PMC9641421; doi:10.15252/emmm.202216283)
Supplement: Supplementary file 6 — Movie EV5 [file EMMM-14-0-s007.zip › EMM-2022-16283-V3-Movie_EV5/Movie EV5.docx]

## Movie EV5. Segmentation of a caseous necrotic lesion with embedded obliterated structures and surrounding vasculature (Sample E).

Caseous necrosis is shown in yellow, vasculature in red. Obliterated structures within the lesions could also be segmented (purple), revealing a branched structure resembling a former airway.
