# Supplementary material for: A high‐resolution 3D atlas of the spectrum of tuberculous and COVID‐19 lung lesions
Source: EMBO Mol Med. 2022 Oct 26;14(11):e16283. doi: 10.15252/emmm.202216283 (PMC9641421; doi:10.15252/emmm.202216283)
Supplement: Supplementary file 7 — Movie EV6 [file EMMM-14-0-s002.zip › EMM-2022-16283-V3-Movie_EV6/Movie EV6.docx]

## Movie EV6. Vasculature in a COVID-19 lung lobe (Sample M).

Maximum intensity projection of µ​CT scan of contrast stained (iodine) lower right lung lobe.
